# Impact of Infection on Survival Outcomes in High-Grade Gliomas: A Retrospective Analysis of 26 Cases in Our Fifteen-Year Experience—*Janus Faced Phenomenon*

**DOI:** 10.3390/cancers17081348

**Published:** 2025-04-17

**Authors:** György Berényi, Dóra Szabó, Gergely Agócs, Blanka Andrássy, Imre Fedorcsák, Loránd Erőss, László Sipos

**Affiliations:** 1 Department of Neurosurgery and Neurointervention, Semmelweis University, 1085 Budapest, Hungarysipos.laszlo.kornel@semmelweis.hu (L.S.); 2 Institute of Medical Microbiology, Semmelweis University, 1089 Budapest, Hungary; 3 Department of Biophysics and Radiation Biology, Semmelweis University, 1094 Budapest, Hungary; 4 HUN-REN-SU Human Microbiota Research Group, 1052 Budapest, Hungary

**Keywords:** glioblastoma IDH-wildtype CNS WHO grade 4, astrocytoma IDH-mutant CNS WHO grade 4, high-grade glioma, surgical site infection, long-term survival, case report

## Abstract

Background/Objectives: Glioblastoma IDH-wildtype CNS WHO grade 4 and astrocytoma IDH-mutant WHO grade 4 (together, high-grade gliomas: HGGs) are the most prevalent malignant brain tumors, carrying a poor prognosis despite multimodal treatment. Surgical site infections (SSIs) represent a relative frequent postoperative complication in HGG patients. Despite multimodal treatment protocols combining surgery, radiotherapy, and temozolomide chemotherapy, HGGs remain associated with a dismal prognosis, underscoring the need to evaluate how SSIs impact disease progression and survival outcomes. This study’s aim was to investigate the influence of SSIs on the clinical course of patients with HGGs. Methods: A comprehensive review of medical records for HGG patients treated at our institution between 2010 and 2024 identified 26 patients with SSIs. These patients were compared to an age-matched control group with the same histological diagnosis and treatment regimen. This study analyzed overall survival (OS), microbiological data, and pathological parameters to assess the impact of SSIs on patient outcomes. Survival differences between the infected and non-infected groups were evaluated using Kaplan–Meier survival curves. Remarkably, three patients with exceptionally long overall survival were highlighted in this study. Results: Among the cohort of 2008 patients with HGG surgery, 26 patients developed SSIs. An age-matched control group of 26 patients was identified, none of whom experienced SSIs. Comparing the OS between the infected and uninfected groups, a statistically significant improvement in OS was observed in the infected group (*p* = 0.049). The median OS in the infected group was 388 days, slightly shorter than the median OS of 422 days in the control group. However, the mean OS was markedly higher in the infected group (674 days) compared to the control group (442 days). The standard deviation of OS in the infected group was notably expansive, indicating substantial variability in survival outcomes. A cluster of infected patients with SSIs near the time of diagnosis had shorter OS, while other infected cases demonstrated significantly longer survival, exceeding both median and mean OS values. In contrast, the uninfected group showed limited standard deviation values, with uniformly distributed individual OS data around the median and mean values. Expectedly, IDH mutation status significantly influenced the survival in cohort patients. However, when stratified by infection status, no association between IDH mutation and improved infection-related survival was identified. The microbiological profile of SSIs was diverse, encompassing Gram-positive and Gram-negative bacteria as well as aerobic and anaerobic organisms. Conclusions: These findings underscore the heterogeneity of infection-related outcomes and their potential impact on survival in HGG patients. According to our knowledge, our study is one of the largest retrospective studies to date investigating and confirming the significant relationship between SSIs and HGG patients’ survival. Our results confirm the Janus Face phenomenon of infections, having both negative and positive effects depending on the context.

## Simple Summary

High-grade gliomas are aggressive brain tumors with poor outcomes, even when treated with surgery, chemotherapy, and radiation. Infections at the site of brain surgery are a known complication, but their effect on survival has been unclear. This study reviewed patients treated between 2010 and 2024 to explore how surgical site infections might influence outcomes. We compared 26 patients who developed infections after surgery to 26 similar patients who did not. Surprisingly, the group with infections showed longer average survival times, although their outcomes varied widely. Some patients with infections lived much longer than expected, while others had shorter survival. In contrast, the patients in non-infected group had similar survival times, but their average survival was shorter. We also found that the bacteria causing the infections varied greatly. A genetic feature commonly linked to better outcomes did not appear to explain the differences in infected patients. This study suggests that infections may have both harmful and potentially helpful effects on survival, depending on timing and other factors. Understanding this unexpected pattern could open new paths for research directions and improved treatment strategies for patients with high-grade gliomas.

## 1. Introduction

The 2021 WHO CNS tumor classification emphasizes molecular features for diagnosis. The CNS WHO grade 4 tumors represent the most aggressive and malignant brain tumors, including glioblastoma IDH-wildtype CNS WHO grade 4 and astrocytoma IDH-mutant CNS WHO grade 4 [1]. In this manuscript, we will refer to both tumors as high-grade gliomas (HGGs). Glioblastomas of IDH-wildtype CNS WHO grade 4 typically occur after age 40 and are characterized by diffuse astrocytic histology without IDH mutations and defined molecular features. The Stupp protocol is the current standard of care for treating glioblastoma IDH-wildtype CNS WHO grade 4. It involves maximal safe surgical resection followed by concurrent radiotherapy and temozolomide chemotherapy, then adjuvant temozolomide cycles [2]. In contrast, astrocytoma IDH-mutant WHO grade 4 represents a distinct entity formerly known as secondary glioblastoma harboring IDH mutations. This tumor generally affects younger adults and have a relatively better prognosis. This group includes both primary and secondary IDH-mutant grade 4 astrocytomas [1].

The failure of the current complex therapy has led to a focus on new therapeutic options, such as immunotherapy [3,4]. Immunotherapy aims to boost the body’s immune response against cancer cells. Interestingly, bacterial infections are known to be immunomodulators in other types of cancer, such as sarcomas and bladder cancer, suggesting that they may activate the immune system in a beneficial way [5,6]. It has been hypothesized that postoperative infections may stimulate the immune system and potentially improve survival in HGG patients. However, this hypothesis is speculative: while some studies suggest that bacterial infections may slow tumor growth through activation of the immune response, other studies have not shown a significant survival benefit after such infections [6,7,8].

In recent years, there has been growing interest in exploring how infections can affect cancer outcomes. For example, certain infections have been associated with improved immune responses in other types of tumors, suggesting that they may also play a role in the treatment of gliomas. The aim of our study is to investigate the impact of clinical infections on the survival of HGG patients treated in our institute over the past 15 years. By analyzing the relationship between postoperative infections and survival outcomes, we hoped to contribute to our understanding of infections could improve the immune response of HGG patients.

## 2. Materials and Methods

### 2.1. Patients Selection and Treatment Protocol

This retrospective study analyzed the clinical data of patients treated at the National Institute of Neuroscience and its successor, the Department of Neurosurgery and Neurointervention, Semmelweis University, Budapest between January 2010 and December 2024. Adult patients diagnosed with glioblastoma IDH-wildtype CNS WHO grade 4 and astrocytoma IDH-mutant CNS WHO grade 4 as high-grade glioma (HGG) during this period by surgical biopsy or tumor resection were included. After surgery, all patients received a standardized treatment consisting of radiotherapy and temozolomide-based chemotherapy according to the Stupp protocol [2].

### 2.2. Data Collection and Patient Cohort

The database contained comprehensive patient data, including demographic characteristics (age and gender), tumor location, histopathological findings (e.g., IDH mutation status), clinical infection data, survival outcomes, and follow-up information. Postoperative infection status was determined based on clinical signs—such as surgical site infections (SSIs), meningitis (SSI-MEN), intracerebral abscesses (SSI-ICs), and craniotomy-related infections (SSI-CRANs)—or positive microbiological findings. In cases where the date of death was not recorded in the hospital records, data were retrieved from publicly available mortality databases using the patient’s social security number.

**Figure 1:**
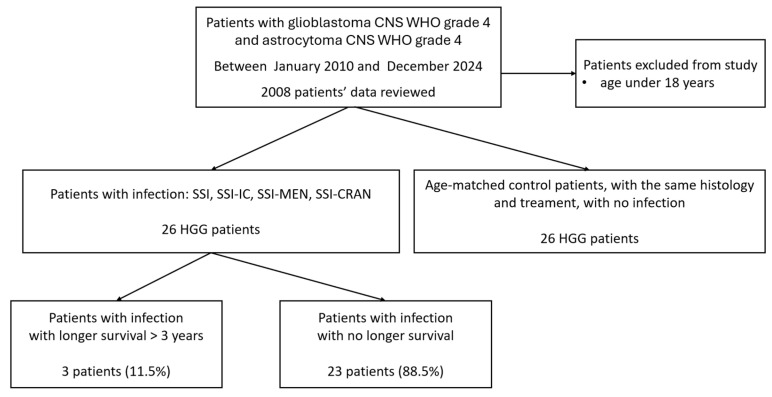
Flowchart of glioblastoma IDH-wildtype CNS WHO grade 4 and astrocytoma IDH-mutant CNS WHO grade 4 patients’ inclusion in this study, survival, and infection status.

A total of 2008 patient records were reviewed for eligibility. Of these, 26 patients were identified as having a confirmed postoperative infection based on microbiological findings and clinical symptoms. The patient selection process is shown in Figure 1. Only individuals with documented surgical site infections were included in the study cohort; patients with non-surgical infections, such as urinary tract or respiratory infections, were excluded due to incomplete data availability and the focus of this research being on surgical site complications.

Matched case–control patients were selected for each of the 26 cases with surgical site infections based on age, sex, histological type, treatment modality and other relevant clinical parameters. Long-term survivors were defined as those achieving an overall survival of more than three years.

### 2.3. Statistical Analysis

The primary endpoint of this study was overall survival (OS), defined as the time from histological diagnosis to death from any cause. Progression-free survival (PFS) was also assessed as a secondary endpoint.

For each infected case, matched control patients based on age, sex, histological type, postoperative treatment protocol, and IDH mutation status were randomly selected from the total patient population. Only patients diagnosed with glioblastoma IDH-wildtype CNS WHO grade 4 and astrocytoma IDH-mutant CNS WHO grade 4 as HGG were included as controls.

Survival analysis was performed using Kaplan–Meier estimates with 95% confidence intervals (CIs). Univariate analysis was performed to examine dichotomous predictors such as infection status, IDH mutation, ATRX expression, and Ki-67 proliferation index. Differences between Kaplan–Meier survival curves were tested using the log-rank test. All statistical analyses were performed in R software (version 4.4.1) [9] using the survival (version 3.8-3) [10] and survminer (version 0.5.0) packages [11].

## 3. Results

### 3.1. Characteristics of the HGG Patients with Infection and Age-Matched Case–Control Population

A total of 2008 patients were diagnosed with HGG between January 2010 and December 2024. The study patient group consisted of 908 women and 1091 men, with a mean age of 61 years. Of these, 26 patients (1.29%) developed clinical infection, with a mean age of 56 years. An age-matched HGG control group was also selected for the infected patients, with a mean age of 56 years.

The clinical characteristics of infected patients were compared with age-matched controls, shown in Table 1. There were no statistically significant differences in age, gender distribution, or anatomical location of tumors between the two groups. The mean age of both groups was 56 years. Overall, 65% of infected cases and 61.5% of controls were male. A similar distribution of tumor localization was observed among the frontal, occipital, temporal, and parietal regions. Regarding postoperative infections, surgical site infections (SSIs), intracerebral abscesses (SSI-ICs), meningitis (SSI-MEN), and craniotomy-related infections (SSI-CRANs) occurred exclusively in the infected group with regard to selection criteria.

### 3.2. The Survival Data of All the Selected 56 Cohort Patients with HGG—The Infected and Uninfected Groups Together

**Figure 2:**
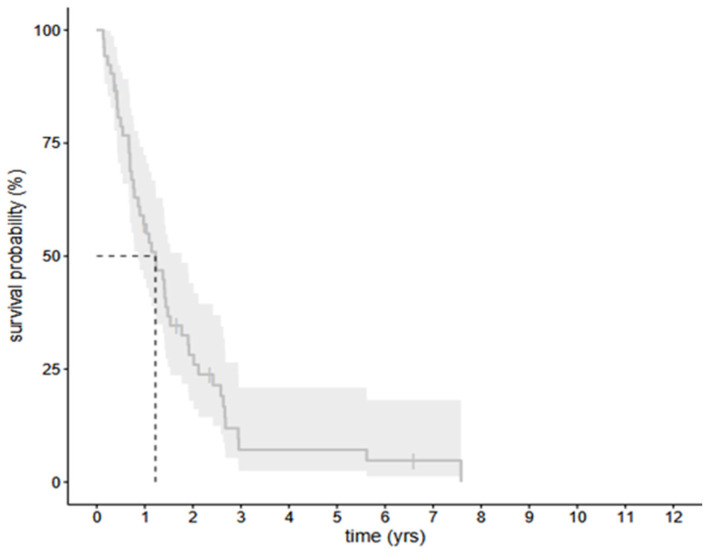
The OS of the all selected 56 patients. Grey areas indicate confidence intervals.

The OS of the selected 56 HGG patients—containing the infected patients and the uninfected case–control patients—is shown in Figure 2, including confidence intervals.

**Figure 3:**
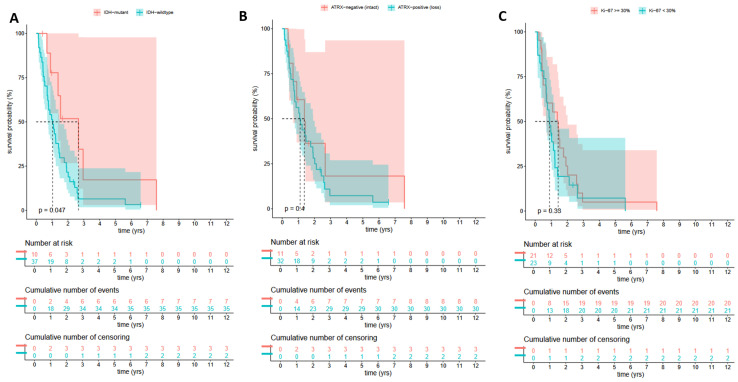
The OS of the selected 56 patients—infected group and non-infected age-matched case–control group—based on IDH mutation status (**A**), ATRX (**B**), and Ki-67 ratio (**C**). Kaplan–Meier functions show a significant difference of survival between patients with (red line) astrocytoma IDH-mutant CNS WHO grade 4 and without mutation (green line) glioblastoma IDH-wildtype CNS WHO grade 4 (**A**); ATRX negative (red line) and ATRX positive (green line) (**B**); Ki-67 ratio ≥ 30% (red line) and ≤30% (red line).

The OS was compared in the all the selected 56 HGG patients in the cohort group—the infected and uninfected groups together—based on histological and pathological parameters—IDH mutation status, ATRX, and Ki-67. The OS was significantly better in patients with astrocytoma IDH-mutant CNS WHO grade 4, regardless of whether they belonged to the infected or uninfected group. However, comparing the OS of the patients based on ATRX status and Ki-67 values, no significant difference was detected (Figure 3).

### 3.3. Comparison of Survival Data Between the Infected and Uninfected Groups of HGG Patients

#### 3.3.1. OS Analysis Between the Infected and Uninfected Groups of HGG Patients

**Figure 4:**
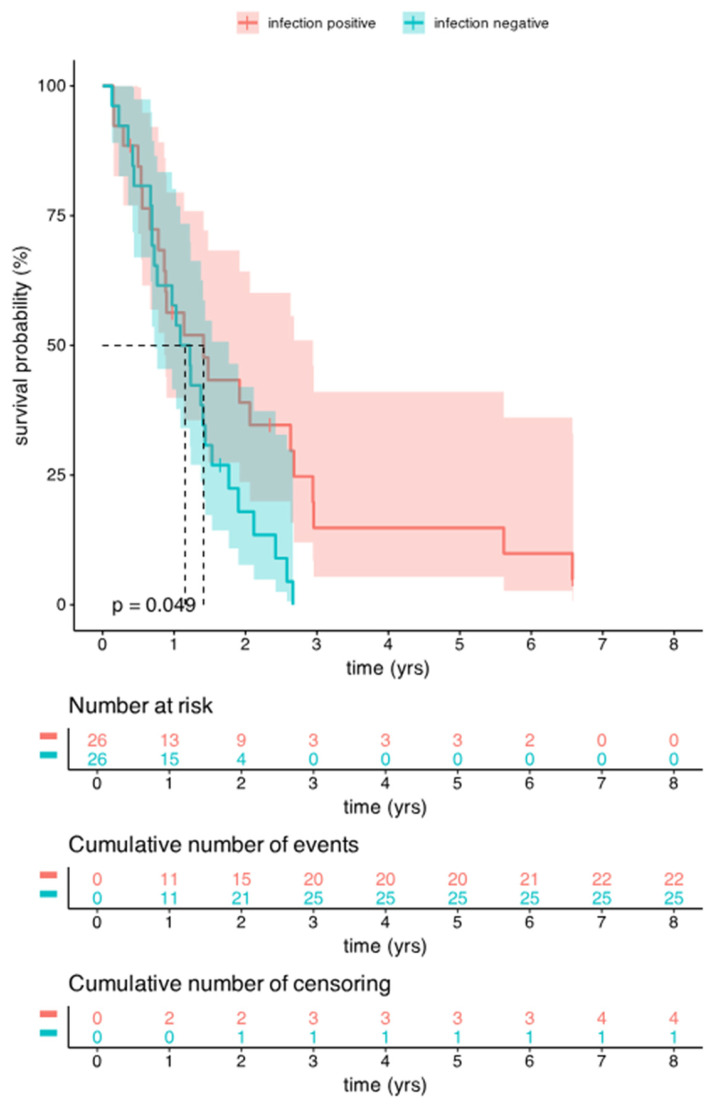
The OS rate of infected (infection positive, red line) and uninfected control groups (infection negative, green line) of HGG patients. Kaplan–Meier survival curves show significant difference (*p* = 0.049) of survival between patients with (red line) clinical infection and control group without infection (green line). Red and green areas indicate the confidence intervals.

The OS was compared in the selected HGG group between the infected and uninfected patients. Remarkably, the OS was significantly (*p* = 0.049) better in the infected group compared to the uninfected case–control group. Figure 4 shows the Kaplan–Meier survival curves of the infected and uninfected control groups. The PFS showed also slight differences, with a median PFS of 343 days in the infected patients and 309 days in the control group (Table 1).

**Figure 5:**
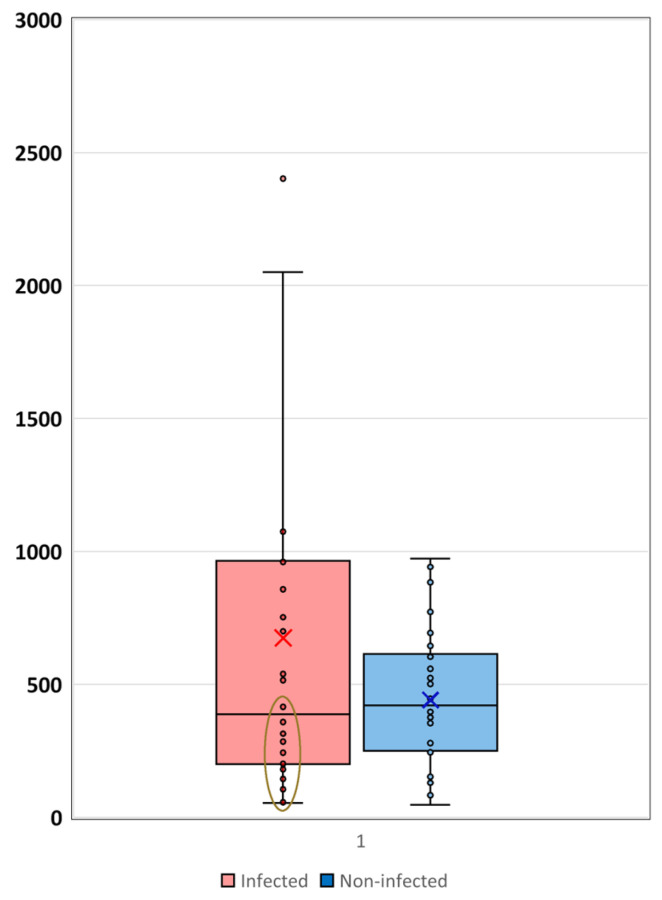
The OS rate with the individual OS data in the infected and uninfected case–control HGG patients’ group. The box plot illustrates the distribution of OS in the infected (red) and in the uninfected case–control (blue) boxes. The central line within each box represents the median OS (50th percentile), while the small cross (X) inside the box indicates the mean OS value. The circle indicates a cluster in individual OS.

Considering the differences in the significant OS values in the infected and uninfected HGG patients, the median and mean and the individual OS values were further analyzed (Figure 5). The median of the OS values was shorter in the infected group (388 days) than in the control group (422 days), and the mean values of OS were also longer in the infected group, with 674 days, in contrast to the 442 days observed in the uninfected group, respectively (Table 1). By analyzing the individual OS values, it is clearly presented in Figure 5 that in the infected group, the standard deviation is very wide and a cluster with a shorter OS was observed close to the diagnosis time (marked with a brown circle), in which cases the OS did not reach either the median or the mean OS values. The remaining infected cases presented much longer OS values (Table 2). In contrast, in the non-infected matched group, the standard deviation of OS was narrower and the median and mean values were much closer to each other. Furthermore, in the uninfected group, the individual OS values showed an even distribution inside the standard deviation values.

#### 3.3.2. OS Analysis Between the Infected and Uninfected HGG Groups Based on Their IDH Mutations Status

**Figure 6:**
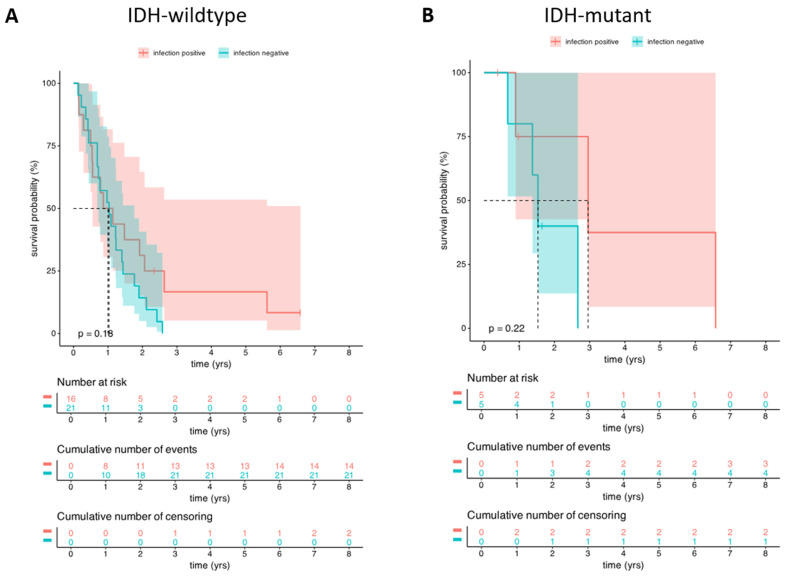
The survival rate of clinical infected and non-infected control groups in the IDH-wildtype (**A**) and IDH-mutant group (**B**) of HGG patients. Kaplan–Meier survivals show no significant difference of survival between patients with (red line) clinical infection and without (green line) clinical infection. Red and green areas indicate the confidence intervals.

Since in the selected cohort HGG population, patients with IDH mutations had significantly better OS, in accordance with the literature data, we examined the OS separately in the IDH-wildtype and IDH-mutant subgroups of the cohort. However, there could not be observed any statistically significant differences in OS between the two groups of patients, but the results might have been influenced by the low sample size. The results are presented in Figure 6.

### 3.4. The Microbiological Results of HGG Patients with Postoperative Infection

Bacteria were cultured from 12 HGG patients’ (46%) wound samples; in eleven cases (42%) no bacteria was detected and in three cases microbiological samples were not taken at all. In seven patients a single bacterium was responsible for the infection and in six out of seven patients the bacteria originated from the skin. In five cases several bacteria together were detected parallelly in the wound infection (Table 2).

*S. aureus* and *S. epidermidis* were cultured in the case of eight patients, at 32% of all infected cases. In three cases *Staphylococcus* spp. were detected with consortium with other bacteria. In two cases methicillin-resistant *S. aureus* strains were cultivated from the wound samples. Gram-positive anaerob bacteria *Actinomyces*, *Peptostreptococcus niger*, and *Cutibacterium acnes*, formerly named *Propionibacterium acnes*, were isolated from three patients. Gram-negative bacteria, namely *Escherichia coli, Enterobacter cloacae, Klebsiella aerogenes,* and *Pseudomonas aeruginosa*, were cultivated from four patients.

In the patients with long survival—more than three years—namely, 2402 days, 2051 days, and 1075 days, the microbiological backgrounds of the infections were very diverse. In one case no bacteria were detected, in one other case *S. aureus* alone was cultured, and in three cases mixed infection occurred with Gram-positive, Gram-negative, and aerobic and anaerobic bacteria together.

#### 3.4.1. Case Reports of Three HGG Patients with Long Survival (>3-Years)

Here, we report three cases with multiple surgical resections, radio-chemotherapy and adjuvant chemotherapy. Despite early tumor progression and post-surgical complications, the patients achieved prolonged disease-free survival. These cases highlight the possible potential benefits and challenges of aggressive surgical management and multimodal therapy in recurrent glioblastoma IDH-wildtype CNS WHO grade 4 and astrocytoma IDH-mutant CNS WHO grade 4.

##### Case Report 1

**Figure 7:**
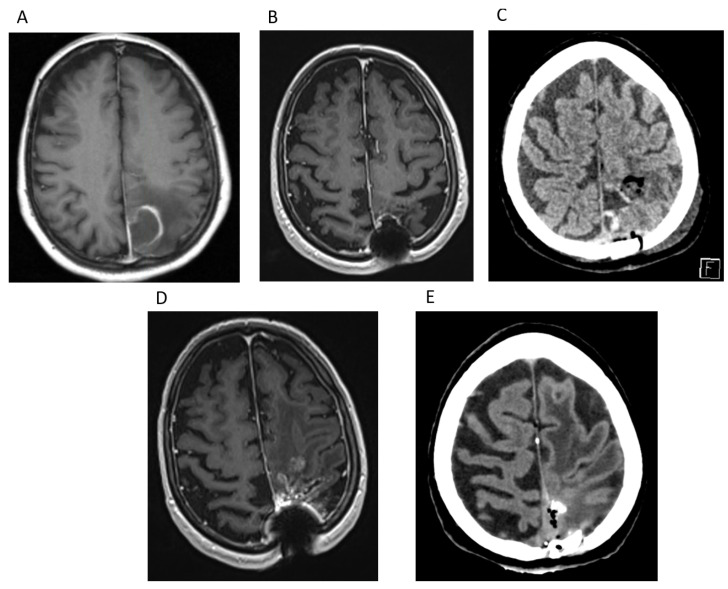
The cranial MRI and CT scan of patient 1. (**A**) Preoperative T1 contrast-weighted axial image; (**B**) postoperative routine T1 contrast-weighted MRI axial image, after chemo-, radiotherapy, no signs of recurrence; (**C**) postoperative routine T1 contrast-weighted MRI axial image. Progression of the lesion with a new cystic component; (**D**,**E**) T1 contrast-weighted MRI axial image and CT axial image obtained at the time of admission. Signs of osteomyelitis were present at the previous surgical site, including bone erosion and air pockets.

A 70-year-old female with a history of treated hypertension and a prior melanoma removal with skin grafting 24 years before presented with neurological symptoms. Preoperative MRI confirmed a left parietal space-occupying lesion (Figure 7A). In 2018, she underwent a left parietal craniotomy with tumor resection at our Institute.

Histopathology confirmed glioblastoma IDH-wildtype CNS WHO grade 4 with immunohistochemistry, showing glial fibrillary acidic protein marker and ATRX positivity but IDH-1 R132H negativity. Postoperatively, she received treatment according to the Stupp protocol. The baseline MRI showed stable conditions with no signs of residual tumor or recurrence (Figure 7B). She completed 18 cycles of temozolomide (TMZ) by 2021, but MRI revealed mild progression of the known parietal-parasagittal lesion with a new cystic component, which was considered disease progression (Figure 7C). TMZ reinduction was offered, but the patient refused it. By late 2024, the patient and her family reported persistent wound discharge from the surgical site for approximately 1.5 years. On arrival in our department, she was alert with right-sided mild hemiparesis. The retracted scalp exposed the skull, emitting a foul odor. After the wound began bleeding, she was transported to the trauma unit and later referred to our department. MRI obtained at the time of admission with contrast and a native CT scan confirmed progression of the recurrent tumor. Additionally, signs of osteomyelitis were present at the previous surgical site, including bone erosion and air pockets (Figure 7D,E). Cultures from the wound revealed *Enterobacter cloacae* and *Peptoniphilus* species. Empirical antibiotic therapy with vancomycin and meropenem was initiated.

Surgical revision was performed, including wound edge refreshment and bone flap removal. A microbiological culture confirmed *E. cloacae*, *Peptococcus niger*, and *Actinomyces turicensis*. According to the neuroinfectologist, vancomycin was discontinued after 10 days and meropenem therapy was continued for another 4 days. Outpatient treatment with levofloxacin 500 mg once daily for 3 weeks was prescribed.

Follow-up and current status: Two months postoperatively, the patient had a 1 mm wound dehiscence with no sign of inflammation or discharge. She remained bedridden with no improvement, so no further neurosurgical interventions were planned.

This case highlights the challenges of managing recurrent glioblastoma, particularly in the context of chronic postoperative complications such as osteomyelitis. Despite tumor progression, the patient survived for over five years post-diagnosis, surpassing the median survival expectations. Her refusal of reinduction chemotherapy may have influenced tumor progression. Additionally, chronic wound infection and osteomyelitis posed significant management challenges, requiring long-term antibiotic therapy and surgical debridement. The OS was 80 months and the PFS was 36 months.

##### Case Report 2

A 30-year-old male patient first presented with neurological symptoms in 2011, leading to the discovery of a space-occupying lesion in the right temporal lobe. The patient underwent a temporal craniotomy and tumor removal was performed. Histopathological analysis confirmed the diagnosis of astrocytoma IDH-mutant CNS WHO grade 4 with ATRX negativity.

**Figure 8:**
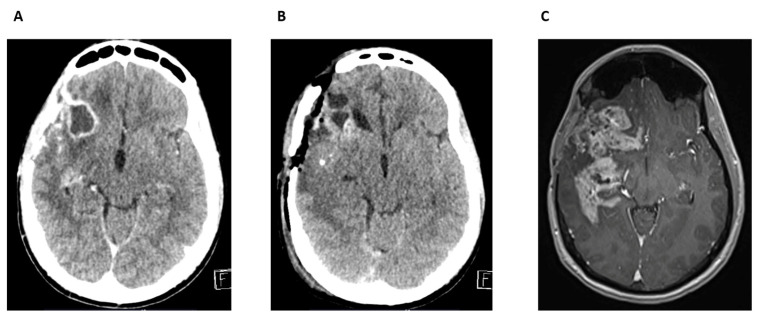
Cranial CT and MRI scan of patient 2. (**A**) Routine follow-up CT scan, axial view. Tumor recurrence in the right temporal region; (**B**) postoperative CT scan, axial view. Signs of infection; (**C**) routine MRI scan, axial slice. Signs of tumor progression.

The patient recovered well postoperatively and subsequently received adjuvant radio-chemotherapy according the Stupp protocol. In 2012, due to tumor progression, reoperation was performed. There were no data available about the further oncological treatment which was performed in a countryside institution. For several years, the patient remained clinically stable. In May 2018, a routine follow-up CT and MRI revealed tumor recurrence in the right temporal region (Figure 8A). The MR scan showed significant perilesional edema displacing the insular cortex and an elongated, calcified lesion along the cortical surface. Based on these findings, the patient underwent a frontotemporal craniotomy in May 2018 for tumor resection. Postoperatively, the patient developed purulent discharge from the surgical wound, raising concerns for infection (Figure 8B). On June 2018, he underwent a third surgical intervention and the infected bone flap was removed. Microbiological analysis confirmed *S. aureus* as the causative pathogen. He was treated with targeted antibiotic therapy, including vancomycin and meropenem. Prolonged antibiotic treatment and careful wound management led to successful infection control.

At a follow-up examination in September 2018, the surgical wound had healed, but the patient’s overall condition had worsened. He exhibited gait instability, somnolence, and progressive neurological decline. A comparative MRI in September 2018 and in April 2018 confirmed further tumor progression (Figure 8C). Multifocal recurrence with extensive contrast enhancement was observed in the right temporal surgical site, with new lesions in the basal ganglia and mesencephalon. The patient was on Avastin therapy. By January 2019, the patient’s condition had significantly worsened and Avastin treatment was discontinued due to his deteriorating clinical status. Best supportive care was recommended.

The OS was 80 months and the PFS was 71 months from the initial surgery in 2011 to the confirmed recurrence in May 2018, highlighting a relatively extended disease course despite the aggressive nature of astrocytoma IDH-mutant CNS WHO grade 4.

##### Case Report 3

**Figure 9:**
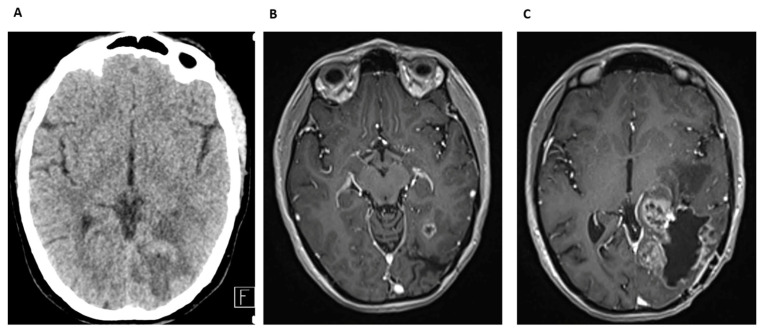
Cranial CT and MRI scan of patient 3. (**A**) Preoperative native CT axial image; (**B**) postoperative routine T1 contrast-weighted axial image; (**C**) postoperative routine T1 contrast-weighted axial image with tumor progression.

A 37-year-old female patient presented to the hospital with hallucinations and persistent headaches lasting for a month. Preoperative CT revealed a left-sided occipital mass measuring 1.5 × 1.6 cm with perifocal edema, though no significant mass effect was observed (Figure 9A). Based on the result of the biopsy, a tumor resection was performed in September 2014. Histopathological examination confirmed glioblastoma IDH-wildtype CNS WHO grade 4, ATRX positivity, and the absence of 1q/19q co-deletion.

Postoperatively, the patient received adjuvant radio-chemotherapy according to the Stupp protocol starting in October 2014. She then continued with 24 cycles of TMZ until October 2016. In 2019, MRI detected a small local recurrence in the left peritrigonal region, characterized by a necrotic center and increased relative cerebral blood volume. This lesion caused compression of the left lateral ventricle with associated edema and a midline shift of 7 mm (Figure 9B). As a result, TMZ was rechallenged for three cycles. Due to further progression, a second surgery was performed in September 2019. Following surgery, Avastin therapy was initiated but had to be discontinued due to postoperative complications such as cerebrospinal fluid leakage, fever, and meningitis. In December 2019, she required a lumbar drain and received antibiotic treatment until January 2020. Despite this, she remained subfebrile in February, with persistent cerebrospinal fluid abnormalities indicating infection.

A follow-up MRI scan revealed another tumor recurrence, leading to a third surgery in February 2020 with concurrent cranioplasty (Figure 9C). By April 2020, she developed progressive headaches, likely from subdural hygroma. A subduroperitoneal shunt relieved her symptoms, but with no further treatment options, she was discharged home for palliative care. The overall OS was 68 months and the patient’s PFS was 60 months.

## 4. Discussion

In the past, infections were a major cause of morbidity and mortality and occurred after almost all operations [12]. Today, this complication occurs in 0.8–7% of patients undergoing craniotomy because antibiotic prophylaxis has reduced the incidence of infections during neurosurgical procedures [13]. The most common infections following neurosurgical procedures occur as meningitis, wound/bone infections, and brain abscesses [14]. Although infections are typically viewed as harmful due to their potential to complicate treatment and increase morbidity, some observations suggest that they may, paradoxically, prolong survival in a subset of HGG patients. This phenomenon is often referred to as the ‘Janus Face’ of infections, having both negative and positive effects depending on the context. It is already known that the presence of IDH mutations can significantly impact the survival and treatment outcomes of HGG patients [7,15,16], as we could confirm as well; however, the impact of postoperative infections on the survival of HGG patients is complex and multifaceted.

Our results showed significant increase in OS of HGG patients with SSIs. While the median OS was slightly shorter in the infected group compared to controls (388 days vs. 422 days), the mean OS was significantly higher (674 days vs. 442 days), suggesting that certain subgroups of infected patients may have prolonged survival. This is in line with observations from previous studies, such as De Bonis et al., which reported a survival benefit in infected patients, particularly those who developed the infection early after surgery [17]. However, conflicting results from other studies, including Salle et al., suggest that infections may lead to shorter survival overall [18]. These differences support the heterogeneity of infection-related outcomes and the need for further stratification based on the timing, type, and severity of infection.

The timing and depth of SSIs appear to be key factors affecting survival outcomes. Late or deep infections, particularly those with intracranial abscesses or bone flap lesions, have been associated with prolonged survival in some studies [6,7,19,20]. Conversely, early infections or superficial wound complications may exacerbate postoperative morbidity without a survival benefit [18]. To date, the evidence in the literature is insufficient and no clear conclusion can be drawn as to whether postoperative infections affect the survival of patients with HGG [8,20], but several anecdotal case reports have been published of patients with a local wound infection after HGG resection and long-term survival [7,8,17,21].

In our study, we also reported three patients with long-term survival who developed infections or demonstrated clinical signs several years after their initial diagnosis. The first case involved a patient who survived more than five years and had a chronic wound infection and osteomyelitis caused by a mixed bacterial infection: *E. cloacae*, *Peptococcus niger*, and *Actinomyces turicensis*. The second case presented a patient with six years of survival following diagnosis and surgery who developed an SSI caused by *S. aureus*. The third case involved a patient who survived six years and later developed meningitis, although no microbiological data were available. This phenomenon has also been observed by others. A case report documented an Ommaya reservoir infection caused by *S. aureus* three years after primary glioblastoma resection, with no tumor recurrence observed for six years following the infection [22]. Similarly, long-term survival in four cases was reported with glioma resection followed by bacterial infection, where the tumor suppression might be enhanced by the immune response and the direct oncolytic effects of bacteria [23]. This hypothesis is further supported by a case report with no glioblastoma recurrence that was observed four years after tumor resection and treatment of the infection caused by *S. epidermidis* [24].

The microbiological analysis of SSIs revealed a diverse spectrum of pathogens, mainly *S. aureus* and *S. epidermidis*, in line with previous reports [25,26,27]. *S. epidermidis* is another common cause of postoperative infections, particularly in patients with implanted devices [28]. *Cutibacterium acne* is often found in cases of epidural infections or abscesses following neurosurgical procedures [29,30,31]. Mixed infections including Gram-positive, Gram-negative, aerobic, and anaerobic bacteria were also observed. *S. aureus* is known for its virulence and ability to cause severe infections. *K. pneumoniae*, though less common, is a gram-negative bacterium that has been identified in some cases of postoperative infections in HGG patients. However, in our cases, other primarily Gram-negative bacteria, mainly Enterobacterales, were also isolated in approximately the same proportion as the more frequently observed bacteria mentioned above. The occurrence of Enterobacterales in neurosurgical SSI is variable [18,32]. The antitumor mechanisms of bacteria are diverse and multifaceted, encompassing direct and indirect approaches to target tumor cells, and can modulate the tumor microenvironment. These mechanisms include stimulating host immune responses, inducing direct cytotoxicity, disrupting cellular signal transduction, remodeling the extracellular matrix, inhibiting neoangiogenesis, and altering tumor metabolism. Some bacteria, including *Klebsiella*, *Listeria*, *Mycobacteria*, *Streptococcus/Serratia* (Coley’s toxin), *Proteus, Salmonella*, and *Clostridium*, have demonstrated direct oncolytic effects in preclinical studies by invading tumor cells and disrupting their metabolic processes [33]. Other bacteria such as *S. aureus* can compete with tumor cells for essential nutrients like iron and glucose [34]; this competition could limit the resources available for tumor growth and angiogenesis, potentially slowing tumor progression.

The immunological impact of these infections may play a critical role in the regulation of tumor progression. Host genetic factors may also influence the response to bacterial infections in HGG patients, particularly through their effects on immune modulation and tumor biology. While direct evidence linking specific genetic factors to bacterial infection responses in HGG is limited, several mechanisms and associations can be inferred. HGG tumors often downregulate MHC class I molecules, impairing antigen presentation and reducing the immune system’s ability to recognize tumor cells [35]. In addition, competition between bacteria and tumor cells for essential nutrients such as iron and glucose may inhibit tumor growth.

However, based on our observations when the infection occurred later, the survival rates were more favorable. In our opinion, in the case of long-term surviving patients, the long-term survival was caused by slow, subclinical infection, since in all presented cases the infection was activated before their death and the activation of the infection also played a role in the death of the patients. In addition, the question arises whether the observed phenomenon is influenced by the type of pathogen or its number of microbes. Since there are many theories regarding the effect of bacteria on the immune system and tumors, it can be assumed that some strains may also produce special metabolites. It is currently not possible to determine the degree of infection or inflammation that may still have a positive effect. Further research is needed to develop indicators that quantify the extent of infection, detect, classify and determine the extent of inapparent infections beyond clinical symptoms, and provide greater insight into the analysis of infections in HGG patients.

Despite some interesting observations of longer survival in some infected patients, SSIs remain a significant burden due to increased hospitalization and longer length of stay. In addition, the negative impact on health-related quality of life should not be overlooked. Clinicians should carefully weigh the potential immunological benefits of SSIs against their adverse effects when managing postoperative complications in HGG patients.

One limitation of this study is that, based on the selection criteria, patients with longer survival and no signs of clinical infection but possible latent infection were not included in the study. However, this does not mean that an in-apparent infection did not exist, only that it had not appeared in clinical form and is therefore “invisible” in this study. Furthermore, for the same reasons, patients who have/had chronic infections affecting other organ systems rather than wound infections could not be included in this study. A further limitation, which was a limitation not only in our study but also in all previous studies, is that unfortunately, further examination of the bacteria (for example by whole genome sequencing) cultured from the infections was not carried out to characterize the properties of the given bacterial strain, such as virulence factors, antigens, metabolic enzymes, etc. An in-depth examination of these bacterial strains may bring us closer to understanding the exact mechanisms involved in maintaining a long latent infection.

## 5. Conclusions

HGG is one of the most aggressive brain tumors, with a poor prognosis despite significant advances in treatment over the past decades. An increasing number of studies are investigating the relationship between postoperative infections and survival, as some observations suggest that infection may stimulate an immune response that could potentially slow tumor progression. According to our knowledge, our study is one of the largest retrospective studies to date investigating and confirming the significant relationship between SSIs and HGG patients’ survival. Infection, however, is a Janus Face phenomenon—while in some cases it may contribute to longer survival by activating the immune system, in other cases it worsens the patient’s condition as a life-threatening complication. The results of a comprehensive fifteen-year study also confirm this contradictory relationship: The current results highlight the necessity to explore the exact mechanisms by which infection exerts its antitumor effect.

## Figures and Tables

**Table 1:** Comparison of the characteristics of HGG patients with infection and age-matched control groups.

Characteristics of the Patients	Patients with Infections (26)	Age-Matched Controls (26)
Mean age (years) (min–max)	56 (30–75)	56 (33–77)
Male (%)	17 (65%)	16 (61.5%)
Localization (%)		
frontal	10 (38%)	9 (35%)
occipital	1 (4%)	1 (4%)
temporal	8 (31%)	10 (38%)
parietal	7 (27%)	6 (23%)
Postoperative infection (%)		
SSI	7 (27%)	0
SSI-IC	13 (50%)	0
SSI-MEN	3 (11.5%)	0
SSI-CRAN	3 (11.5%)	0
PFS (days)		
mean	343	309
median	179	259
OS (days)		
mean	674	442
median	388	422

SSI, SSI-IC, SSI-MEN, SSI-CRAN.

**Table 2:** The characteristics of infected HGG patients.

Patients	Age at Diagnosis (Years)	Localization	Reoperation	IDH-Type	ATRX	Ki-67 %	PFS (Days)	OS (Days)	Infection Type	Time Elapsed After Surgery and Infection (Days)	Microbiological Results
**1**	50	temporal	yes	IDH-wild	neg	45	80	202	SSI-MEN	5	*Escherichia coli*, *Enterococcus faecalis*
**2**	56	frontal	no	IDH-wild	pos	30	176	539	SSI-IC	170	ND
**3**	60	frontal	yes	IDH-wild	pos	22.5	54	54	SSI-IC	21	negative
**4**	49	temporal	yes	ND	ND	ND	378	1075	SSI	954	*Staphylococcus aureus*
**5**	73	temporal	yes	IDH-wild	pos	30	147	753	SSI-IC	202	*Staphylococcus epidermidis*
**6**	53	temporal	yes	IDH-wild	pos	20	39	416	SSI-IC	15	ND
**7**	57	parietal	yes	IDH-mutant	neg	25	327	327	SSI-IC	217	*Klebsiella aerogenes*
**8**	60	parietal	yes	IDH-wild	pos	ND	234	285	SSI-IC	68	negative
**9**	51	frontal	yes	IDH-wild	neg	15	182	322	SSI-IC	217	negative
**10**	68	parietal	yes	ND	pos	55	162	181	SSI-IC	45	*Propionibacterium acnes*
**11**	65	frontal	yes	IDH-wild	neg	20	57	57	SSI-IC	25	negative
**12**	67	frontal	yes	IDH-wild	pos	27.5	27	197	SSI-IC	102	*Staphylococcus epidermidis*, *Cutibacterium acnes*
**13**	64	parietal	yes	IDH-wild	pos	ND	1096	2406	SSI-IC	2356	*Enterobacter cloacae*, *Peptoniphilus species*, *Peptococcus niger Actinomyces turicensis*
**14**	56	parietal	yes	ND	ND	20	173	243	SSI	229	ND
**15**	48	parietal	yes	ND	ND	ND	516	516	SSI-CRAN	487	*Methicillin resistant Staphylococcus aureus*, *Pseudomonas aeruginosa*
**16**	53	parietal	yes	IDH-wild	pos	15	60	315	SSI-CRAN	68	negative
**17**	58	frontal	no	IDH-wild	pos	25	289	961	SSI	14	negative
**18**	57	temporal	yes	IDH-wild	pos	40	462	700	SSI-IC	484	*Staphylococcus epidermidis*
**19**	30	frontal	ND	IDH-mutant	neg	40	2116	2402	SSI-CRAN	2175	*Staphylococcus aureus*
**20**	75	temporal	yes	ND	ND	ND	950	978	SSI	96	*Methicillin-resistant Staphylococcus aureus*
**21**	63	temporal	no	IDH-wild	pos	22.5	106	106	SSI	35	negative
**22**	35	frontal	no	IDH-mutant	pos	45	933	1079	SSI	6	negative
**23**	37	occipital	no	IDH-wild	pos	25	1818	2051	SSI	1943	negative
**24**	48	frontal	yes	IDH-wild	pos	22.5	702	858	SSI-IC	33	negative
**25**	52	temporal	yes	IDH-mutant	neg	17.5	359	359	SSI-MEN	10	*Enterococcus faecalis*, *Staphylococcus epidermidis*
**26**	37	frontal	yes	IDH-mutant	neg	55	145	145	SSI-MEN	20	negative

ND: no data, PFS: progression free survival, OS: overall survival.

## Data Availability

The datasets used and/or analyzed during the current study are available from the corresponding author.

## Abbreviations

The following abbreviations are used in this manuscript:

GBM	GBM
KPS	Karnofsky Performance Score
WHO	Word Health Organization
IDH	isocitrate dehydrogenase
OS	overall survival
PFS	progression free survival
SSI	surgical site infection
SSI-MEN	surgical site infection—meningitis
SSI-IC	surgical site infection—intracranial abscess
SSI-CRAN	surgical site infection—craniotomy
ATRX	Alpha-thalassemia X-linked mutant retardation syndrome
TMZ	temozolomide
CT	computed tomography
MRI	Magnetic Resonance Imaging
ASA	acetyl salicylic acid
HGG	high-grade glioma
AA	anaplastic astrocytoma
MHC	major histocompatibility complex
